# Evaluation of MCL-1 as a prognostic factor in canine mammary gland tumors

**DOI:** 10.1371/journal.pone.0306398

**Published:** 2024-07-16

**Authors:** Jaeho Cho, Heaji Chung, Sungin Lee, Wan Hee Kim

**Affiliations:** 1 Department of Veterinary Clinical Sciences, College of Veterinary Medicine and Research Institute for Veterinary Science, Seoul National University, Seoul, Republic of Korea; 2 Department of Veterinary Surgery, College of Veterinary Medicine, Chungbuk National University, Cheongju, Republic of Korea; University of Colorado Denver School of Medicine, UNITED STATES OF AMERICA

## Abstract

Myeloid cell leukemia-1 (MCL-1), which belongs to the anti-apoptotic B cell lymphoma-2 family protein, is overexpressed in various cancers and is associated with cell immortality, malignant transformation, chemoresistance, and poor prognosis in humans. However, the significance of MCL-1 in canine mammary gland tumors (MGTs) remains unknown. This study aimed to examine MCL-1 expression in normal canine mammary glands and tumors and to assess its correlation with clinical and histologic variables. In total, 111 samples were examined, including 12 normal mammary gland tissues, 51 benign MGTs, and 48 malignant MGTs. Immunohistochemistry revealed that 53% of benign tumors and 75% of malignant tumors exhibited high MCL-1 expression, whereas only 8% of normal mammary glands exhibited high MCL-1 expression. High MCL-1 expression correlated with tumor malignancy (p < 0.001), large tumor size (> 3 cm) (p = 0.005), high Ki-67 expression (p = 0.046), and metastasis (p = 0.027). Survival curve analysis of dogs with malignant MGTs demonstrated a significant association between high MCL-1 expression and shorter median overall survival (p = 0.027) and progression-free survival (p = 0.014). Our study identified MCL-1 as a prognostic factor and potential therapeutic target in canine MGTs.

## Introduction

Myeloid cell leukemia-1 (MCL-1), a member of the B-cell lymphoma-2 (BCL-2) protein family, was initially discovered during the differentiation of human myeloid leukemia cells into monocytes or macrophages with phorbol 12-myristate 13-acetate (TPA) exposure [1]. It is known to influence the development and progression of various tumors [2]. In addition to its association with tumorigenesis, MCL-1 is implicated in physiologically essential roles, such as fetal development and mitochondrial homeostasis [3]. Mitochondria-dependent apoptosis, which is regulated by BCL-2 family member proteins, involves anti-apoptotic proteins, pro-apoptotic pore formers, and pro-apoptotic BH3-only proteins. Oligomerization of pro-apoptotic and anti-apoptotic proteins results in mitochondrial outer membrane permeabilization (MOMP), which causes the release of apoptogenic factors, such as cytochrome c, from the mitochondrial intermembrane space into the cytosol. APAF1 and procaspase-9 assemble to form apoptosomes. Subsequent activation of procaspase-9 initiates apoptosis [4]. Among the proteins regulating this series of apoptosis events, MCL-1, which belongs to an anti-apoptotic group, inhibits cell death by preventing the oligomerization of BAX and BAK effector proteins, which trigger apoptosis [3]. Previous findings support the notion that suppression of MCL-1 expression leads to increase BAK protein expression and accelerates cell apoptosis [5].

The term "tumor" refers to an abnormally growing mass that destructively impacts the surrounding normal tissues due to excessive autonomous growth. Therefore, the association of MCL-1 with tumors is a natural consequence of its mechanism. Numerous studies have reported that high MCL-1 expression is linked to the occurrence, progression, metastasis, and poor prognosis of various tumors, including human breast, renal, hepatocellular, gastric, pulmonary, ovarian cancers, and lymphoma [2, 6–12]. Specifically, in human breast cancer, a study using MMTV-PyMT mice revealed high MCL-1 expression in both primary mammary tumors and metastatic lesions. Deletion of MCL-1 in MMTV-PyMT mice demonstrated its essential role of MCL-1 in the tumorigenesis of mammary gland tumors (MGTs) [2]. Several selective MCL-1 inhibitors (BH3 mimetics) that target the BH3 domain-binding groove are currently undergoing clinical trials as a novel therapy [3].

MGTs are the most common tumors in intact female dogs, with research indicating that approximately 11.1% to 53.3% of intact female dogs may have malignant MGTs [13, 14]. These tumors typically occur in older dogs and are commonly reported in toy and miniature Poodles, Spaniel breeds, Maltese, Yorkshire terriers, Dachshunds, Doberman Pinschers, and German Shepherd dogs. Histologic type, tumor size, various factors (Ki-67 and AgNORs), lymph node metastasis, and distant metastasis play crucial roles in determining the prognosis of canine MGTs [15]. The primary treatment option is surgical excision, which is considered the ideal method for achieving adequate local control unless there is a suspicion of distant metastasis or inflammatory mammary carcinoma, where the likelihood of prognostic improvement, even with excision, is significantly low. Adjuvant therapies such as radiation therapy, endocrine therapy, and chemotherapy are available, however, effective regimens have not yet been determined, and their use in canine MGTs is limited [16].

In humans, extensive research has been conducted on the prognostic value and therapeutic utility of MCL-1 expression in various tumors. Therefore, exploring MCL-1 as a prognostic factor in veterinary medicine is valuable. To the best of our knowledge, there has been no prior research on the correlation of MCL-1 expression with various variables or prognostic evaluation in canine MGTs, except for studies evaluating MCL-1 messenger RNA (mRNA) expression in canine MGTs using real-time PCR [17]. A previous study demonstrated a reduction in MCL-1 mRNA expression and an increase in the apoptotic rate of canine MGT cell lines through the application of small interfering RNA (siRNA) [18]. In this study, we investigated the expression levels of MCL-1 in normal mammary tissues, benign mammary tumors, and malignant mammary tumors using immunohistochemistry. Additionally, correlations between MCL-1 expression and various clinical and histologic variables were analyzed to assess the potential of MCL-1 as a prognostic factor. We hope this study will lay the foundation for new prognostic factor and treatment strategies for canine MGTs.

## Materials and methods

### Tissue samples and clinical data

Ninety-nine dogs that underwent surgery at Seoul National University Veterinary Hospital between August 2019 and June 2023 were included in the study. The owners of dogs with MGT were informed about the procedure and purpose of this study, and the informed consent was written and obtained. On August 1, 2023, the data of these individuals were accessed for the purpose of the study. During and after the data collection process, access of individual information regarding the collected subjects were not permitted. To alleviate the suffering of the dogs with MGT, 22 mg/kg IV Cefazolin was given 30 minutes prior to surgery, and the dogs were premedicated with a remifentanil-midazolam-ketamine constant rate infusion (CRI) (remifentanil 0.11–0.22 μg/kg/min IV CRI, midazolam 0.02–0.04 mg/kg/hr IV CRI, ketamine 0.375–0.75 mg/kg/hr IV CRI). General anesthesia was induced with propofol 6 mg/kg IV and maintained with isoflurane. To alleviate pain during surgery, the remifentanil-midazolam-ketamine CRI was administrated and adjusted based on pain response and vital signs. MGT tissues were aseptically collected from these dogs, and histopathological examinations were conducted using hematoxylin and eosin (H&E) staining, either at the IDEXX laboratory or the Seoul National University Veterinary Pathology Laboratory. Of these samples, 51 were identified as benign and 48 as malignant tumors. The MGT tissues were classified into different histologic types based on the diagnostic criteria outlined in the 2011 Goldschmidt paper [19]. The tissues were fixed in 10% neutral buffered formalin for 48 h at 15–20°C and embedded in paraffin blocks for immunohistochemical staining. In cases where an individual dog had more than one mammary tumor, the more aggressive tumor was selected for classification, following the recommendations of Sorenmo [20].

All patients with MGTs underwent preoperative physical examinations, serum chemistry analyses, complete blood counts, radiographic examinations, and abdominal ultrasound examinations to exclude other malignancies and to determine the clinical stage. Clinical data, tumor size, histologic type, and information regarding metastasis were collected. All experimental procedures adhered to ethical standards and were approved by the Seoul National University Institutional Animal Care and Use Committee (IACUC) under reference numbers SNU-201202-3 and SNU-231206-3.

Normal mammary gland tissues were obtained from twelve 2-year-old, intact female beagles housed in the Department of Veterinary Surgery, College of Veterinary Medicine, Seoul National University. These dogs (SNU-200709-4-5 and SNU-181214-3) were unrelated to any experiments related to MGTs and used in different studies. Before euthanasia, they underwent a thorough physical examination, serum chemistry analysis, complete blood count, radiographic examination, and abdominal ultrasonography to confirm their overall health. None of the dogs had a history of pregnancy, and vaginal cytology confirmed their anestrus status. To alleviate pain, the dogs were premedicated with tramadol 4 mg/kg IV as an analgesic and acepromazine 10 μg/kg IV as a sedative. General anesthesia was induced with alfaxalone 2–3 mg/kg IV and maintained with isoflurane. After mammary gland tissue collection, an additional alfaxalone 2 mg/kg IV was administered, followed by a KCl solution 10 ml IV bolus for euthanasia. Euthanasia was confirmed by auscultation. Mammary gland tissues were aseptically collected immediately after euthanasia and the collected tissues were fixed and embedded. Histopathological examination, following H&E staining, confirmed that the tissues were normal mammary glands.

### Immunohistochemistry

The paraffin-embedded canine mammary gland tissues were fixed in 10% neutral buffered formalin and sectioned into 3-μm-thick sections. These sections were placed in a 60°C incubator, followed by deparaffinization through a series of xylene and graded ethanol (100%, 100%, 90%, 80%, 70%, PBS for 5 min each). Antigen retrieval was performed using a 10 mM citric acid buffer (pH 6.0) in a 2100-retriever pressure cooker (PickCell Laboratories, Amsterdam, Netherlands) for 20 min. The tissues were treated with a peroxidase-blocking solution (3% H_2_O_2_) for 30 min to block endogenous peroxidase activity. To prevent nonspecific reactions, sections were blocked with normal goat serum (ab7481; Abcam, Waltham, MA, USA) at 15–20°C for 20 min. Incubation with rabbit anti-MCL-1 polyclonal antibody (1:500, Ab28147; Abcam, Waltham, MA, USA) was carried out overnight at 4°C. The sections were then incubated at 15–20°C for 10 min with a biotinylated secondary antibody (biotinylated goat anti-rabbit IgG (H+L) (ready to use), ab64256, Abcam, Waltham, MA, USA) followed by incubation at 15–20°C for 10 min with streptavidin-horseradish peroxidase (HRP) (ab64269; Abcam, Waltham, MA, USA). To visualize the antigen, the sections were developed at 15–20°C for 5 min using 3,3’-diaminobenzidine (DAB Substrate kit, ab64238, Abcam, Waltham, MA, USA). The process of washing with phosphate-buffered saline was performed between all the previously described steps. Tissue sections were counterstained with Mayer’s hematoxylin, dehydrated with graded ethanol, washed with xylene, and rinsed with phosphate-buffered saline during the intermediate steps.

For Ki-67 staining, rabbit anti-Ki-67 polyclonal primary antibody (1:500, PA5-19462; Invitrogen) was incubated at 15–20°C for 1 h, followed by HRP goat anti-rabbit IgG (H + L) (1:1000; 32460, Invitrogen, Paisley, UK) secondary antibody at 15–20°C for 1 h. Based on the literature, we used Leydig cells from mouse testicular tissue as a positive control for immunohistochemical staining with MCL-1. This tissue was selected because Leydig cells demonstrate a strong tendency to stain when MCL-1 is used as previously reported [21].

To eliminate the nonspecific binding of the secondary antibody, normal mammary gland tissue was stained without the primary antibody and used as a negative control. The stained slides were scanned using an Olympus BX50F4 microscope (Olympus, Tokyo, Japan) equipped with a light filter (Tucsen, Fuzhou, China).

### Quantification of immunohistochemistry staining

Two observers (Jaeho Cho and Heaji Chung) independently observed the cells and analyzed their scores as follows: expression intensity was scored as 0 (negative), 1 (weak), 2 (moderate), or 3 (strong). A minimum of five representative fields at 400× magnification and over 1,000 cells were observed to calculate the percentage of stained cells, which resulted in scores ranging from 0 to 100 based on the ratio. Finally, the histoscore was calculated by multiplying the expression intensity score by the expression ratio score and summing them. To set an appropriate cut-off value, we plotted the receiver operating characteristic (ROC) curve and calculated sensitivity and specificity using the corresponding data. Based on the intersection point of the sensitivity and specificity curves, the cut-off value was set at 184 to classify MCL-1 expression as high or low. And a similar approach was used for Ki-67 staining analysis. A minimum of five representative fields at 400× magnification and over 1,000 cells were observed to determine the ratio of cells with stained nuclei. Following the guidelines of Kadthur, a cut-off value of 15% was set to distinguish between high and low Ki-67 expression [22]. The total histoscore was independently evaluated by two observers (Jaeho Cho and Heaji Chung) who were blinded to the other observer’s histoscore. Inter-observer agreement was measured using the inter-class correlation coefficient, and ICCC value was confirmed to be > 0.9.

### Follow-up data

All dogs underwent regular check-ups two weeks after surgery and afterward at 3–6-month intervals. Physical examination, thoracic radiography (three-dimensional views), abdominal ultrasound, fine-needle aspiration, and, when necessary, biopsy or CT scans were performed to assess tumor recurrence and metastasis. Additionally, survival status, cause of death, metastasis, and recurrence were confirmed via telephone interviews with the dog’s owners to analyze the survival periods in the malignant MGT group.

### Statistical analysis

All statistical analyses were conducted using SPSS software (IBM Corp., Armonk, NY, USA). The correlation between MCL-1 expression and mammary gland tissue type (normal mammary gland tissue, benign MGT tissue, and malignant MGT tissue), as well as the correlation between MCL-1 expression and the histologic grades of malignant MGTs, were analyzed using the linear-by-linear association test. The correlation between MCL-1 expression and other clinical and histologic variables was analyzed using Fisher’s exact and chi-square tests. The correlation between MCL-1 expression and age was assessed using the Student’s t-test, while the correlation with variables such as metastasis, histologic classification, and Ki-67 expression was analyzed using the Kolmogorov—Smirnov and Shapiro—Wilk tests.

Considering only the group with malignant tumor tissues, Kaplan—Meier survival curves were plotted, and comparisons were made using the log-rank test for survival analysis. Overall survival (OS) was defined as the time from the date of surgery to death. Disease-free survival (DFS) was defined as the time from the date of surgery to the first confirmed metastasis, local recurrence, or death. In the OS study, data were censored for cases in which follow-up was discontinued, those that remained alive at the end of the study, and those in which death occurred for reasons unrelated to malignant MGTs. In the DFS study, data were censored for cases in which the follow-up was discontinued, those who remained alive without metastasis or recurrence until the end of the study, or those who died without metastasis or recurrence. Statistical significance was set at p < 0.05.

## Results

### Clinical and histologic information

Data for all 111 dogs included in the study, including age, sex, breed, and histologic types of tumors, are presented in Table 1. Histologic types were classified according to Goldschmidt’s criteria [19]. Maltese, toy poodles and Yorkshire terriers were the most commonly affected breeds.

**Table 1:** Clinical and histologic variables of the dogs included in this study.

	Normal mammary gland N = 12	Benign tumor N = 51	Malignant tumor N = 48
**Median age (range)**	2	11 (5–16)	11 (6–15)
**Sex (n)**	Spayed female (0)	Spayed female (22)	Spayed female (19)
	Female (12)	Female (29)	Female (29)
**Breed (n)**	Beagle (12)	Maltese (16)	Maltese (11)
		Yorkshire terrier (8)	Toy poodle (10)
		Cocker spaniel (5)	Shih-tzu (6)
		Mixed (5)	Cocker spaniel (4)
		Shih-tzu (4)	Mixed (3)
		Toy poodle (3)	Pomeranian (3)
		Miniature pinscher (2)	Chihuahua (2)
		Pomeranian (2)	Dachshund (2)
		Alaskan malamute (1)	Yorkshire terrier (2)
		Beagle (1)	Bichon frise (1)
		Boxer (1)	Japanese spitz (1)
		Chihuahua (1)	Jindo dog (1)
		Samoyed (1)	Malinois (1)
		Schnauzer (1)	Schnauzer (1)
**Histologic type**	-	Complex adenoma (19)	Carcinoma, simple (23)
		Adenoma, simple (16)	Carcinoma, complex type (11)
		Benign mixed tumor (11)	Carcinoma, mixed type (7)
		Intraductal papillary adenoma (4)	Carcinoma arising in a mixed tumor (2)
		Ductal adenoma (1)	Adenosquamous carcinoma (1)
			Carcinosarcoma (1)
			Carcinoma, solid (1)
			Ductal carcinoma (1)
			Malignant myoepithelioma (1)
**Histologic grade**	-	-	Grade I (27)
	-	-	Grade II (13)
	-	-	Grade III (8)

### MCL-1 expression in normal mammary gland tissue and mammary gland tumors

**Fig 1:**
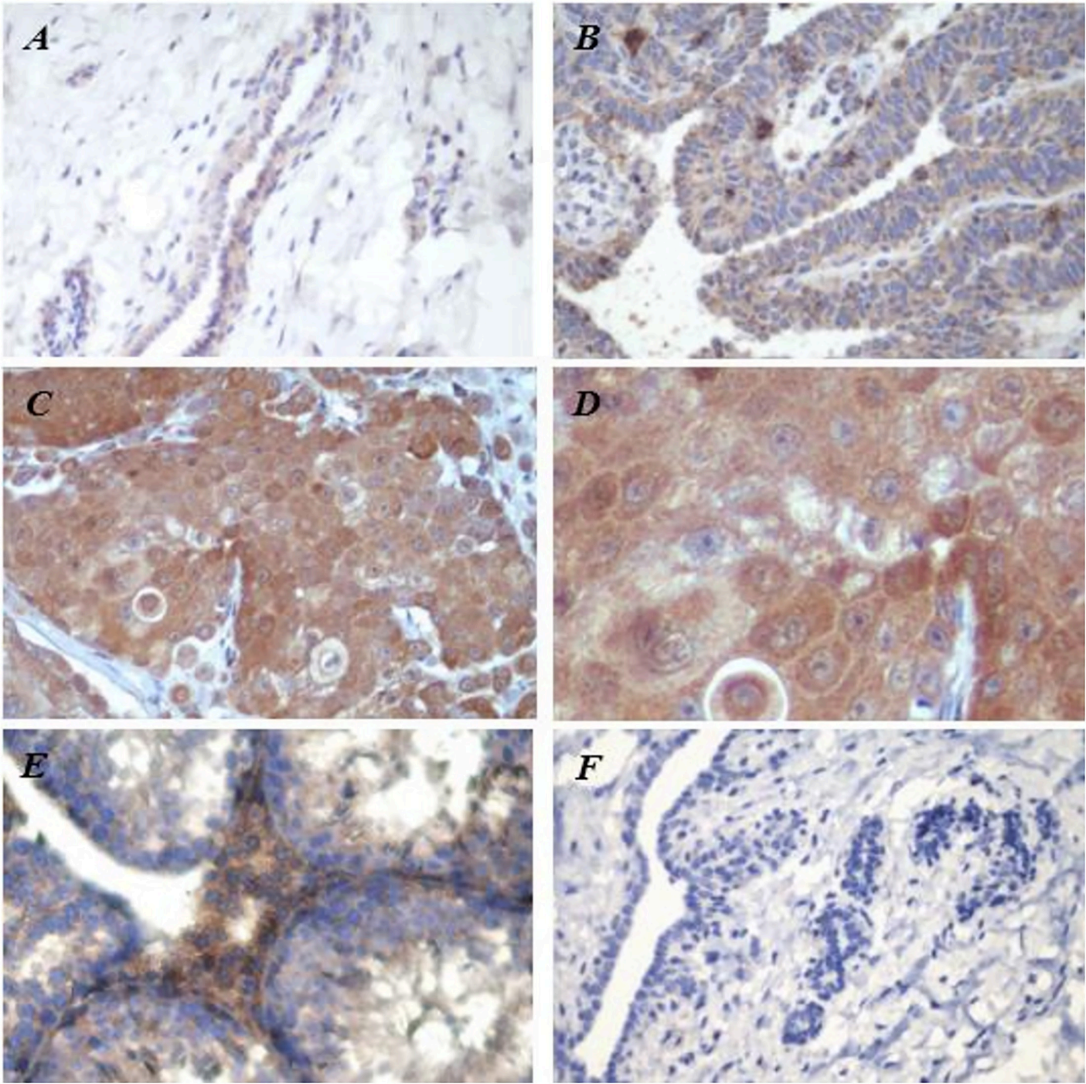
(**A**) MCL-1 immunohistochemistry in canine normal mammary gland. (**B**) Benign mammary gland tumor showing low MCL-1 expression. (**C, D**) Malignant mammary gland tumor showing high MCL-1 expression. (**E**) In the positive control, MCL-1 expression was abundantly observed at Leydig cells in the mouse testis. **(F)** No specific staining was observed in the negative control (A, B, C, E, F original magnification ×400; D, original magnification ×1000).

Immunohistochemical staining of MCL-1 predominantly occurred in the cytoplasm of mammary epithelial cells. As the malignancy of the mammary gland tissues increased from normal to malignant tumors, the intensity of MCL-1 expression increased (Fig 1A–1D). The percentage of high MCL-1 expression increased significantly with tumor malignancy, with rates of 8%, 53%, and 75% in normal, benign, and malignant mammary gland tissues, respectively (Table 2). As the malignancy of mammary gland tissue increases, a significant increase in the proportion of high MCL-1 expression occurs.

**Table 2:** MCL-1 expression in normal mammary glands, benign MGTs, and malignant MGTs.

	Normal mammary gland N = 12 N (%)	Benign tumor N = 51 N (%)	Malignant tumor N = 48 N (%)	P-value
**MCL-1 expression**				
**Low**	11 (92)	24 (47)	12 (25)	p < 0.001 for low vs high *
**High**	1 (8)	27 (53)	36 (75)	

* P-value < 0.05, indicating a statistically significant linear association between tumor malignancy and high MCL-1 expression.

### Correlation of clinical and histologic variables and MCL-1 expression

Table 3 shows the correlation between clinical and histologic variables and MCL-1 expression. The median age of the high- and low-expression groups was 11 years. No significant correlation was observed between the histologic type of MGTs and MCL-1 expression. Tumors > 3 cm were significantly associated with high MCL-1 expression, which is consistent with previous findings linking larger tumors to poor prognosis [23]. In malignant MGTs, high Ki-67 expression and metastasis were significantly associated with high MCL-1 expression. However, no significant correlation was observed between the histologic grade of the malignant MGTs and MCL-1 expression (Table 4).

**Table 3:** Clinical and histologic variables associated with MCL-1 expression level.

	MCL-1 expression			
	Number of tumors	Low	High	P-value
**Median age (range) (years)**		11 (6–16)	11 (5–16)	0.557
**Tumor size (n)**				
≤ 3cm	68	31	37	
> 3cm	31	5	26	**0.005** *
**Benign tumor (n)**				
Simple	16	7	9	
Complex	19	8	11	
Mixed	11	7	4	
Intraductal papillary adenoma	4	1	3	
Ductal adenoma	1	1	0	0.497
**Malignant tumor (n)**				
Simple	23	4	19	
Complex	11	1	10	
Mixed	7	3	4	
Carcinoma arising in a mixed tumor	2	1	1	
Adenosquamous	1	0	1	
Carcinosarcoma	1	1	0	
Carcinoma, solid	1	0	1	
Ductal carcinoma	1	1	0	
Malignant myoepithelioma	1	1	0	0.089
**Ki-67 (n)**				
≤ 15%	24	9	15	
> 15%	24	3	21	**0.046** *
**Metastasis (n)**				
No	26	10	16	
Yes	15	1	14	**0.027** *

* A p-value < 0.05, indicating a statistically significant association between high MCL-1 expression and clinical and histologic variables.

**Table 4:** MCL-1 expression according to the histologic grade of malignant MGTs.

	Malignant tumor, grade 1 N = 27 N (%)	Malignant tumor, grade II N = 13 N (%)	Malignant tumor, grade III N = 8 N (%)	P-value
**MCL-1 expression**				
**Low**	10 (37)	2 (15)	0 (0)	p = 0.067 for low vs high
**High**	17 (63)	11 (85)	8 (100)	

### Survival curve

**Fig 2:**
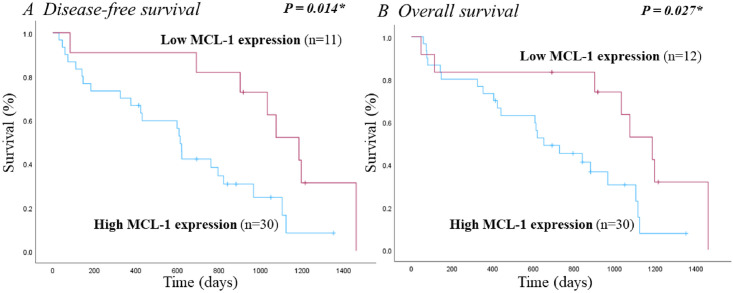
Kaplan—Meier survival curve of disease-free survival (A) and overall survival (B) based on MCL-1 expression of 42 dogs with malignant MGTs. (1 dog was excluded from the disease-free survival statistics because metastasis was confirmed at the time of surgery).

Among the 48 patients with malignant mammary tumors, 6 who discontinued follow-up were excluded from both the OS and DFS analyses. Additionally, 1 patient with confirmed pulmonary metastasis at the time of surgery was excluded from the disease-free survival analysis. Therefore, OS was analyzed in 42 dogs, and DFS was analyzed in 41 dogs using Kaplan—Meier analysis. In the OS study, 30 dogs were in the high MCL-1 expression group, while 12 were in the low MCL-1 expression group. That 1 excluded from the DFS analysis owing to confirmed pulmonary metastasis at the time of surgery belonged to the low MCL-1 expression group. Among the 42 dogs included in the OS study, 18 died from MGTs and 13 from unrelated causes. In the OS analysis, eight dogs in the high MCL-1 expression group and three dogs in the low MCL-1 expression group were right-censored, indicating that they were still alive at the end of the study period. In the DFS analysis during the study period, 16 dogs in the high MCL-1 expression group experienced recurrence or metastasis, whereas only 1 dog in the low MCL-1 expression group experienced metastasis. The survival curve revealed that the high MCL-1 expression group had significantly poorer disease-free survival and overall survival. The median overall survival time for the high MCL-1 expression group was 652 days and the median disease-free survival time was 613 days (Fig 2).

## Discussion

In this study, we aimed to assess the expression of MCL-1 in normal mammary gland tissues using immunohistochemistry and to investigate the differences in MCL-1 expression between normal mammary glands and MGTs. In humans, immunohistochemistry has been used to evaluate the expression levels of MCL-1 in various normal tissues, including the skin, gastrointestinal tract, endocrine system, nervous system, lymphatic system, cardiovascular system, respiratory system, and breast [24]. Similar research has been conducted in dogs as well [25]. A previous study suggested variations in apoptotic development in normal mammary gland tissues based on the estrous cycle [26]. This study used anestrus samples from normal beagle dogs as the normal group based on a reference study.

While the exact role of MCL-1 in mammary gland tissue is not clearly defined, previous studies in rodents have suggested that MCL-1 is essential for the survival of mammary epithelial cells during developmental processes such as the expansion of ductal trees and alveoli, thereby emphasizing its importance in mammary gland development [27]. In this study, using canine mammary gland tissues, MCL-1 was found to exhibit a predominantly cytoplasmic staining pattern in mammary epithelial cells. MCL-1 primarily localizes to the mitochondrial membrane, inhibiting the oligomerization of BAX and BAK proteins and thereby interfering with the apoptosis process [28].

This study confirmed that MCL-1 expression in normal mammary gland tissue was significantly lower, and malignant tumor tissue exhibited higher MCL-1 expression than benign tumor tissue. Considering the gradual progression of benign to malignant tumors in dogs and the higher MCL-1 expression in invasive ductal carcinoma than in normal human tissues, the observed pattern of increased malignancy correlating with higher MCL-1 expression was not unexpected [29]. Moreover, findings from human breast cancer study using the MMTV-PyMT mouse model highlighted the crucial role of MCL-1 in early tumor development and progression. The correlation between MCL-1 expression and stemness markers, as well as increased sensitivity to anticancer drugs and the inability to sustain tumors upon MCL-1 silencing, further supports its significance [2, 30, 31]. Considering these findings, the observed pattern of increased MCL-1 expression with higher malignancy appears natural. Similar mechanisms are anticipated in animals and warrant further biological investigations.

Analysis of the relationship among MCL-1 expression, survival, and metastasis revealed statistically significant correlations. High MCL-1 expression was associated with shorter overall survival (OST; p = 0.027), disease-free survival (DFS; p = 0.014), and the presence of metastasis (p = 0.027). This suggests that, independent of traditional prognostic indicators, high MCL-1 expression itself may serve as a prognostic factor. Previous human studies reported that high MCL-1 expression is associated with shorter survival times and poor outcomes in breast cancer, ovarian carcinoma, acute myeloid leukemia, and chronic lymphocytic leukemia [2, 10, 32, 33]. Furthermore, this study confirmed a significant association between high MCL-1 expression and previously considered clinical indicators of poor prognosis, such as Ki-67 levels exceeding 15% and tumor size larger than 3 cm. Based on these findings, MCL-1 may be considered as a novel prognostic factor for canine MGTs.

The histologic grade of malignant MGTs is generally known to be a poor prognostic factor. Table 4 shows a gradual increase in the proportion of tumors with high MCL-1 expression from histologic grades 1 to 3, suggesting a correlation between malignancy and high MCL-1 expression; however, statistically, a significant correlation was not observed. Considering that MCL-1 is an anti-apoptotic protein inhibiting apoptosis, and all malignant tumors may exhibit abnormal cell proliferation and reduced apoptosis, the relevance of MCL-1 to histologic grade might be low. A previous veterinary study analyzing the mRNA expression of MCL-1 based on the type and grade of MGTs reported no correlation between histologic grade and high MCL-1 expression; however, that study had limitations, such as a small sample size for grade 3 malignant MGTs [17]. Given the observed trend of increasing high MCL-1 expression with higher grades in this study and considering the similarity to Namita’s study where a limited number of high-grade tumor samples were available, further research with a larger sample size remains necessary.

This study also has some limitations. First, we did not perform real-time PCR or western blotting, hence this study lacked quantitative confirmation of mRNA or protein levels. Additionally, all beagles used to obtain normal tissues were intact females under two years of age and the tissues were collected during anestrus. In contrast, patients with MGT underwent surgery after the estrus phase, when the mammary glands regress, to determine the precise surgical dose. However, owing to the retrospective nature of the study that focused on patients, controlling the phase of the estrous cycle was challenging, making it difficult to control for differences associated with the estrous cycle in MCL-1 expression. Further studies on the differences in MCL-1 expression in each phase of the estrous cycle in dogs are required. Another limitation is the relatively small sample size of dogs with histologic grades 2 and 3 MGTs compared with the other groups. Future studies should address these limitations when investigating MCL-1 expression in canine MGTs and normal mammary gland tissues. Considering the differences in protein expression between animal species and variations in antibody staining efficiency across species, it can be noted as a final limitation that mouse Leydig cells were used as the positive control. However, no research has shown a significantly stronger expression in dog Leydig cells than in other cells, and this was not evaluated in our study. We hope that dog Leydig cells can be evaluated as a positive control for the MCL-1 protein in future research.

## Conclusion

This study investigated the expression of MCL-1 in normal canine mammary gland tissues and MGTs using immunohistochemistry. These findings revealed significantly lower MCL-1 expression in normal tissues, whereas malignant tumor tissues exhibited higher MCL-1 expression than benign tumor tissues. The high MCL-1 expression group demonstrated shorter disease-free and overall survival times and was statistically associated with tumor size, Ki-67 expression, and metastasis. These results suggest that MCL-1 could serve as a prognostic and diagnostic marker.

## Supporting information

S1 Table Clinical and pathological variables and immunohistochemistry scores of the dogs used in this study. (DOCX)
