# Supplementary material for: Evaluation of MCL-1 as a prognostic factor in canine mammary gland tumors
Source: PLoS One. 2024 Jul 16;19(7):e0306398. doi: 10.1371/journal.pone.0306398 (PMC11251587; doi:10.1371/journal.pone.0306398)
Supplement: S1 Table — (DOCX) [file pone.0306398.s001.docx]

S1 Table. Clinical and pathological variables immunohistochemistry scores of the dogs used in this study

|  | Species | Sex | Age | Tumor size | Histopathology | Grade | IHC MCL-1 | IHC MCL-1 | IHC Ki-67 | IHC Ki-67 | Metastasis | Recurrence | Survival time | Disease free interval |
| --- | --- | --- | --- | --- | --- | --- | --- | --- | --- | --- | --- | --- | --- | --- |
| Normal 1 | Beagle | Female | 2 | - | - | - | 0 | Low | - | - | - | - | - | - |
| Normal 2 | Beagle | Female | 2 | - | - | - | 0.625 | Low | - | - | - | - | - | - |
| Normal 3 | Beagle | Female | 2 | - | - | - | 1 | Low | - | - | - | - | - | - |
| Normal 4 | Beagle | Female | 2 | - | - | - | 1 | Low | - | - | - | - | - | - |
| Normal 5 | Beagle | Female | 2 | - | - | - | 1.125 | Low | - | - | - | - | - | - |
| Normal 6 | Beagle | Female | 2 | - | - | - | 1.35 | Low | - | - | - | - | - | - |
| Normal 7 | Beagle | Female | 2 | - | - | - | 1.375 | Low | - | - | - | - | - | - |
| Normal 8 | Beagle | Female | 2 | - | - | - | 1.5 | Low | - | - | - | - | - | - |
| Normal 9 | Beagle | Female | 2 | - | - | - | 1.625 | Low | - | - | - | - | - | - |
| Normal 10 | Beagle | Female | 2 | - | - | - | 1.75 | Low | - | - | - | - | - | - |
| Normal 11 | Beagle | Female | 2 | - | - | - | 1.8 | Low | - | - | - | - | - | - |
| Normal 12 | Beagle | Female | 2 | - | - | - | 1.875 | High | - | - | - | - | - | - |
| Benign 1 | Schnauzer | Spayed female | 11 | ≤3 cm | Adenoma, simple | - | 1.625 | Low | - | - | - | - | - | - |
| Benign 2 | Yorkshire Terrier | Female | 9 | ≤3 cm | Benign mixed tumor | - | 1.375 | Low | - | - | - | - | - | - |
| Benign 3 | Shih-tzu | Female | 9 | ≤3 cm | Complex adenoma | - | 1.625 | Low | - | - | - | - | - | - |
| Benign 4 | Yorkshire Terrier | Spayed female | 10 | ≤3 cm | Complex adenoma | - | 0 | Low | - | - | - | - | - | - |
| Benign 5 | Mixed | Spayed female | 10 | ≤3 cm | Complex adenoma | - | 1.575 | Low | - | - | - | - | - | - |
| Benign 6 | Maltese | Female | 14 | ≤3 cm | Benign mixed tumor | - | 1.625 | Low | - | - | - | - | - | - |
| Benign 7 | Shih-tzu | Female | 15 | ≤3 cm | Adenoma, simple | - | 0 | Low | - | - | - | - | - | - |
| Benign 8 | Poodle | Spayed female | 14 | ≤3 cm | Benign mixsed tumor | - | 0.875 | Low | - | - | - | - | - | - |
| Benign 9 | Maltese | Spayed female | 11 | ≤3 cm | Adenoma, simple | - | 1.625 | Low | - | - | - | - | - | - |
| Benign 10 | Alaskan malamute | Female | 8 | ≤3 cm | Complex adenoma | - | 1.65 | Low | - | - | - | - | - | - |
| Benign 11 | Maltese | Female | 11 | ≤3 cm | Adenoma, simple | - | 1.5 | Low | - | - | - | - | - | - |
| Benign 12 | Poodle | Female | 7 | ≤3 cm | Complex adenoma | - | 1.125 | Low | - | - | - | - | - | - |
| Benign 13 | Cocker spaniel | Female | 13 | ≤3 cm | Adenoma, simple | - | 1.5 | Low | - | - | - | - | - | - |
| Benign 14 | Maltese | Spayed female | 13 | ≤3 cm | Adenoma, simple | - | 1.5 | Low | - | - | - | - | - | - |
| Benign 15 | Maltese | Female | 12 | ≤3 cm | Complex adenoma | - | 1.35 | Low | - | - | - | - | - | - |
| Benign 16 | Maltese | Spayed female | 10 | ≤3 cm | Intraductal papillary adenoma | - | 1.5 | Low | - | - | - | - | - | - |
| Benign 17 | Maltese | Spayed female | 10 | ≤3 cm | Benign mixed tumor | - | 1.475 | Low | - | - | - | - | - | - |
| Benign 18 | Maltese | Female | 6 | ≤3 cm | Benign mixed tumor | - | 1.375 | Low | - | - | - | - | - | - |
| Benign 19 | Cocker spaniel | Spayed female | 11 | ≤3 cm | Adenoma, simple | - | 1.375 | Low | - | - | - | - | - | - |
| Benign 20 | Yorkshire Terrier | Female | 10 | ≤3 cm | Adenoma, simple | - | 2.2 | High | - | - | - | - | - | - |
| Benign 21 | Yorkshire Terrier | Spayed female | 12 | ≤3 cm | Complex adenoma | - | 2.25 | High | - | - | - | - | - | - |
| Benign 22 | Cocker Spaniel | Female | 6 | ≤3 cm | Adenoma, simple | - | 2.583 | High | - | - | - | - | - | - |
| Benign 23 | Mixed | Female | 8 | ≤3 cm | Adenoma, simple | - | 2.45 | High | - | - | - | - | - | - |
| Benign 24 | Miniature Pinscher | Female | 9 | ≤3 cm | Benign mixed tumor | - | 2.125 | High | - | - | - | - | - | - |
| Benign 25 | Yorkshire Terrier | Female | 10 | ≤3 cm | Adenoma, simple | - | 2.125 | High | - | - | - | - | - | - |
| Benign 26 | Yorkshire Terrier | Spayed female | 16 | ≤3 cm | Complex adenoma | - | 2.125 | High | - | - | - | - | - | - |
| Benign 27 | Yorkshire Terrier | Female | 14 | ≤3 cm | Complex adenoma | - | 2.875 | High | - | - | - | - | - | - |
| Benign 28 | Yorkshire Terrier | Female | 15 | ≤3 cm | Complex adenoma | - | 1.75 | Low | - | - | - | - | - | - |
| Benign 29 | Maltese | Spayed female | 13 | >3 cm | Complex adenoma | - | 2.375 | High | - | - | - | - | - | - |
| Benign 30 | Mixed | Female | 16 | ≤3 cm | Benign mixed tumor | - | 1.833 | Low | - | - | - | - | - | - |
| Benign 31 | Maltese | Female | 14 | ≤3 cm | Benign mixed tumor | - | 2.45 | High | - | - | - | - | - | - |
| Benign 32 | Mixsed | Female | 10 | ≤3 cm | Benign mixed tumor | - | 1.75 | Low | - | - | - | - | - | - |
| Benign 33 | Maltese | Female | 8 | ≤3 cm | Complex adenoma | - | 2.25 | High | - | - | - | - | - | - |
| Benign 34 | Maltese | Female | 9 | ≤3 cm | Benign mixed tumor | - | 2.375 | High | - | - | - | -- | - | - |
| Benign 35 | Pomeranian | Spayed female | 10 | ≤3 cm | Intraductal papillary adenoma | - | 2 | High | - | - | - | - | - | - |
| Benign 36 | Cocker Spaniel | Spayed female | 9 | ≤3 cm | Adenoma, simple | - | 2 | High | - | - | - | - | - | - |
| Benign 37 | Beagle | Female | 13 | ≤3 cm | Complex adenoma | - | 2.125 | High | - | - | - | - | - | - |
| Benign 38 | Poodle | Female | 7 | ≤3 cm | Complex adenoma | - | 2.15 | High | - | - | - | - | - | - |
| Benign 39 | Samoyed | Female | 8 | ≤3 cm | Benign mixed tumor | - | 2.125 | High | - | - | - | - | - | - |
| Benign 40 | Cocker Spaniel | Spayed female | 9 | ≤3 cm | Adenoma, simple | - | 2.2 | High | - | - | - | - | - | - |
| Benign 41 | Maltese | Female | 5 | ≤3 cm | Complex adenoma | - | 2.125 | High | - | - | - | - | - | - |
| Benign 42 | Maltese | Female | 7 | ≤3 cm | Complex adenoma | - | 2.625 | High | - | - | - | - | - | - |
| Benign 43 | Mixed | Spayed female | 16 | ≤3 cm | Intraductal papillary adenoma | - | 2.25 | High | - | - | - | - | - | - |
| Benign 44 | Boxer | Spayed female | 8 | ≤3 cm | Complex adenoma | - | 2.625 | High | - | - | - | - | - | - |
| Benign 45 | Maltese | Female | 13 | ≤3 cm | Adenoma, simple | - | 2.2 | High | - | - | - | - | - | - |
| Benign 46 | Shih-tzu | Spayed female | 11 | ≤3 cm | Ductal adenoma | - | 1.7 | Low | - | - | - | - | - | - |
| Benign 47 | Maltese | Spayed female | 9 | ≤3 cm | Complex adenoma | - | 2 | High | - | - | - | - | - | - |
| Benign 48 | Shih-tzu | Spayed female | 11 | ≤3 cm | Adenoma, simple | - | 3 | High | - | - | - | - | - | - |
| Benign 49 | Pomeranian | Spayed female | 11 | >3 cm | Intraductal papillary adenoma | - | 2.275 | High | - | - | - | - | - | - |
| Benign 50 | Miniature pinscher | Spayed female | 14 | ≤3 cm | Complex adenoma | - | 1.75 | Low | - | - | - | - | - | - |
| Benign 51 | Chihuahua | Female | 12 | ≤3 cm | Adenoma, simple | - | 2.5 | High | - | - | - | - | - | - |
| Malignant 1 | Jindo | Spayed female | 13 | >3 cm | Carcinoma, mixed type | 1 | 1.083 | Low | 11 | Low | - | - | 1460 | - |
| Malignant 2 | Cocker spaniel | Spayed female | 11 | >3 cm | Carcinoma, simple | 1 | 1.5 | Low | 4.4 | Low | - | - | 1460 | - |
| Malignant 3 | Shih-tzu | Female | 15 | >3 cm | Carcinoma, mixed type | 2 | 1.5 | Low | 27.5 | High | - | - | 902 | - |
| Malignant 4 | Poodle | Female | 12 | >3 cm | Carcinoma, mixed type | 1 | 1.625 | Low | 7.6 | Low | - | - | 1185 | - |
| Malignant 5 | Dachshund | Spayed female | 12 | ≤3 cm | Carcinoma, simple | 2 | 1 | Low | 43 | High | - | - | 1075 | - |
| Malignant 6 | Pomeranian | Female | 12 | ≤3 cm | Ductal carcinoma | 1 | 1.25 | Low | 3.8 | Low | - | - | 1215+ | - |
| Malignant 7 | Poodle | Female | 7 | ≤3 cm | Carcinoma, simple | 1 | 1.5 | Low | 5.4 | Low | + | - | 114 | 83 |
| Malignant 8 | Maltese | Female | 7 | ≤3 cm | Carcinoma, simple | 1 | 1.25 | Low | 3.4 | Low | - | - | 917+ | - |
| Malignant 9 | Maltese | Female | 13 | ≤3 cm | Malignant myoepithelioma | 1 | 1.5 | Low | 7.6 | Low | - | - | 691+ | - |
| Malignant 10 | Chihuahua | Female | 13 | ≤3 cm | Carcinoma arising in a mixed tumor | 1 | 1.5 | Low | 14.4 | Low | + | - | 47 | 0 |
| Malignant 11 | Maltese | Female | 13 | >3 cm | Carcinoma, complex type | 1 | 1.167 | Low | 2.4 | Low | - | - | 1196 | - |
| Malignant 12 | Chihuahua | Female | 14 | ≤3 cm | Carcinoma, simple | 1 | 2.625 | High | 18 | High | - | + | 353 | 141 |
| Malignant 13 | Yorkshire terrier | Female | 13 | >3 cm | Carcinoma, mixed type | 2 | 2.875 | High | 16.6 | High | - | - | 325 | - |
| Malignant 14 | Maltese | Spayed female | 9 | >3 cm | Carcinoma, simple | 1 | 3 | High | 8.4 | Low | - | - | 1123 | - |
| Malignant 15 | Maltese | Spayed female | 6 | >3 cm | Carcinoma, complex type | 1 | 2.25 | High | 12.8 | Low | + | - | 441 | 430 |
| Malignant 16 | Marinoise | Female | 12 | >3 cm | Carcinoma, complex type | 2 | 3 | High | 1.2 | Low | + | - | 608 | 608 |
| Malignant 17 | Poodle | Female | 9 | ≤3 cm | Carcinoma, complex type | 1 | 3 | High | 15.2 | High | + | - | 881 | 822 |
| Malignant 18 | Shih-tzu | Female | 10 | >3 cm | Carcinoma, mixed type | 3 | 2.875 | High | 36.8 | High | + | - | 146 | 146 |
| Malignant 19 | Maltese | Female | 10 | >3 cm | Carcinoma, mixed type | 1 | 2 | High | 14 | Low | - | - | - | - |
| Malignant 20 | Poodle | Female | 9 | >3 cm | Carcinoma, complex type | 1 | 1.85 | High | 4.6 | Low | + | - | 841 | 761 |
| Malignant 21 | Yorkshire terrier | Female | 15 | >3 cm | Carcinoma, complex type | 2 | 2.875 | High | 15.8 | High | + | - | 620 | 620 |
| Malignant 22 | Maltese | Spayed female | 12 | >3 cm | Carcinoma, complex type | 3 | 3 | High | 19.4 | High | + | - | 729 | 183 |
| Malignant 23 | Mixed | Female | 15 | >3 cm | Adenosquamous carcinoma | 3 | 2.75 | High | 19.6 | High | - | - | 1104 | - |
| Malignant 24 | Poodle | Female | 10 | >3 cm | Carcinoma, simple | 3 | 2.875 | High | 15.6 | High | + | + | 142 | 111 |
| Malignant 25 | Maltese | Spayed female | 11 | >3 cm | Carcinoma, complex type | 1 | 2.25 | High | 9.4 | Low | - | - | 966 | - |
| Malignant 26 | Dachshund | Female | 14 | >3 cm | Carcinoma, complex type | 1 | 2.875 | High | 5.2 | Low | - | - | - | - |
| Malignant 27 | Schnauzer | Female | 13 | ≤3 cm | Carcinosarcoma | 1 | 1.75 | Low | 15.2 | High | - | - | 1033 | - |
| Malignant 28 | Poodle | Spayed female | 14 | >3 cm | Carcinoma, simple | 3 | 2.75 | High | 22.6 | High | - | + | 1115 | 598 |
| Malignant 29 | Cocker spaniel | Spayed female | 10 | ≤3 cm | Carcinoma, simple | 3 | 2.75 | High | 18.8 | High | + | - | 73 | 73 |
| Malignant 30 | Shih-tzu | Spayed female | 15 | >3 cm | Carcinoma, simple | 2 | 2 | High | 16.8 | High | + | - | 405 | 376 |
| Malignant 31 | Poodle | Female | 14 | >3 cm | Carcinoma, simple | 1 | 2.75 | High | 10 | Low | + | - | 652 | 621 |
| Malignant 32 | Cocker spaniel | Spayed female | 10 | >3 cm | Carcinoma, simple | 3 | 2.875 | High | 18.2 | High | + | - | 59 | 59 |
| Malignant 33 | Japanese spitz | Spayed female | 9 | ≤3 cm | Carcinoma, simple | 1 | 2.4 | High | 12.4 | Low | - | - | 1351+ | - |
| Malignant 34 | Shih-tzu | Female | 11 | >3 cm | Carcinoma, simple | 1 | 2.75 | High | 9 | Low | - | - | 1050+ | - |
| Malignant 35 | Mixed | Female | 8 | >3 cm | Carcinoma, simple | 2 | 3 | High | 19 | High | - | - | 882+ | - |
| Malignant 36 | Poodle | Female | 11 | >3 cm | Carcinoma, simple | 1 | 2.875 | High | 21.6 | High | - | - | 841+ | - |
| Malignant 37 | Poodle | Female | 13 | >3 cm | Carcinoma, simple | 3 | 2.75 | High | 15.6 | High | + | - | 76 | 46 |
| Malignant 38 | Cocker spaniel | Female | 13 | ≤3 cm | Carcinoma, simple | 1 | 2.25 | High | 7.4 | Low | - | - | 423 | - |
| Malignant 39 | Poodle | Spayed female | 15 | ≤3 cm | Carcinoma arising in a mixed tumor | 1 | 2.875 | High | 15.6 | High | - | - | 692+ | - |
| Malignant 40 | Maltese | Female | 13 | ≤3 cm | Carcinoma, simple | 1 | 2 | High | 9.8 | :Low | - | - | 613 | - |
| Malignant 41 | Bichon frise | Female | 9 | ≤3 cm | Carcinoma, simple | 1 | 3 | High | 11.4 | Low | - | - | 414+ | - |
| Malignant 42 | Mixed | Spayed female | 8 | >3 cm | Carcinoma, simple | 2 | 2.875 | High | 15.6 | High | - | - | 882+ | - |
| Malignant 43 | Shih-tzu | Spayed female | 11 | ≤3 cm | Carcinoma, complex type | 1 | 2.875 | High | 0.4 | Low | - | - | 795+ | - |
| Malignant 44 | Pomeranian | Spayed female | 9 | ≤3 cm | Carcinoma, solid | 2 | 3 | High | 3.4 | Low | + | - | 80 | 30 |
| Malignant 45 | Maltese | Female | 10 | >3 cm | Carcinoma, simple | 2 | 2 | High | 20.4 | High | - | - | - | - |
| Malignant 46 | Shih-tzu | Spayed female | 11 | >3 cm | Carcinoma, mixed type | 2 | 1.85 | High | 18.6 | High | - | - | - | - |
| Malignant 47 | Pomeranian | Spayed female | 10 | ≤3 cm | Carcinoma, complex type | 2 | 1.9 | High | 16.8 | High | - | - | - | - |
| Malignant 48 | Maltese | Spayed female | 9 | ≤3 cm | Carcinoma, simple | 2 | 1.875 | High | 17.8 | High | - | - | - | - |
